# Targeting mTOR and DNA repair pathways in residual triple negative breast cancer post neoadjuvant chemotherapy

**DOI:** 10.1038/s41598-020-80081-y

**Published:** 2021-01-08

**Authors:** Kartik Anand, Tejal Patel, Polly Niravath, Angel Rodriguez, Jorge Darcourt, Anna Belcheva, Toniva Boone, Joe Ensor, Jenny Chang

**Affiliations:** 1 Houston Methodist Cancer Center/Weill Cornell Medicine, OPC 24, 6445 Main Street, Houston, TX 77030 USA; 2 Houston Methodist Research Institute, Houston, TX 77030 USA

**Keywords:** Breast cancer, Cancer therapeutic resistance

## Abstract

Triple-negative breast cancer (TNBC) patients who do not achieve pathologic complete response post neoadjuvant chemotherapy have a poor prognosis. Alteration in PI3K/mTOR plus DNA repair pathways are some of the major mechanisms of chemotherapy resistance. We designed an open-label phase II clinical trial to evaluate if the combination of everolimus (mTOR inhibitor) plus cisplatin (interferes with DNA function) will improve the rate of pathologic response, as assessed by residual cancer burden (RCB). Twenty-four Stage II/III TNBC patients with residual cancer > 1 cm post neoadjuvant anthracycline and taxane-based chemotherapy were enrolled. Patients received everolimus daily orally at 10 mg for 12 weeks and cisplatin IV at 20 mg/m^2^ weekly for 4 cycles (21-day cycle), until definitive surgery. The primary endpoint was the rate of RCB-0-I at the surgery. The median age of the whole cohort was 50.1 years, with 66.7% non-Hispanic Caucasians. Of the 24 patients enrolled, 22 were included in the efficacy analysis. Twenty-one patients underwent definitive surgery while one patient developed distant metastasis. Five patients had RCB-I at surgery, a response rate of 23% (5/22). Patients with germline PALB2 mutation or somatic PI3KCA mutation had a pathologic response, achieving RCB-I at the surgery. Three patients had metaplastic histology achieving RCB-I at the surgery. Estimated OS at 1 year was 100% in the RCB-I group vs. 76.5% in others, which was not statistically significant due to the small sample size. Certain cohorts including PALB2 germline mutation carrier and somatic PI3KCA mutations warrant further investigation.

**Trial registration**: Clinicaltrials.gov identifier: NCT01931163. https://clinicaltrials.gov/ct2/show/NCT01931163.

## Introduction

Triple-negative breast cancers (TNBC) are characterized by lack of expression of estrogen receptor (ER), progesterone receptor (PR), and human epidermal growth factor receptor 2 (HER-2)^1^. TNBC occurs in 10–20% of all breast cancers and has a poor prognosis^1,2^. Patients who do not achieve pathological complete response (pCR) to neoadjuvant chemotherapy have worse survival compared to patients who have pCR at surgery, with more than 30% of these patients relapsing within 3 years^3,4^. Thus, there remains a need to improve outcomes for TNBC who have residual disease post neoadjuvant chemotherapy. Sporadic TNBC and germline mutated BRCA 1/2 associated cancer are both typically basal-like by gene expression profiling^5^. Since BRCA1 and BRCA2 dysfunction leads to impaired homologous recombination repair of DNA^6^, drugs targeting DNA repair is a way to improve outcomes in TNBC. Cisplatin which targets DNA function has previously shown clinical efficacy in the neoadjuvant setting in TNBC as a single agent^7^. Alterations in Phosphoinositide 3-kinase/mammalian target of rapamycin (PI3K/mTOR) pathway followed by DNA repair alterations have been described as the major mechanisms of chemotherapy resistance in TNBC patients with residual disease post neoadjuvant chemotherapy^8^. Thus, we designed a rational Phase II trial of combination everolimus which targets the PI3K/mTOR pathway by inhibiting mTOR, and cisplatin which targets DNA function in the neoadjuvant setting in TNBC patients who have residual disease post anthracycline or taxane-based chemotherapy. The primary objective of the trial was the rate of pathologic response, as measured by the Residual Cancer Burden (RCB) after treatment with cisplatin plus everolimus.

## Results

### Patient characteristics

**Figure 1:**
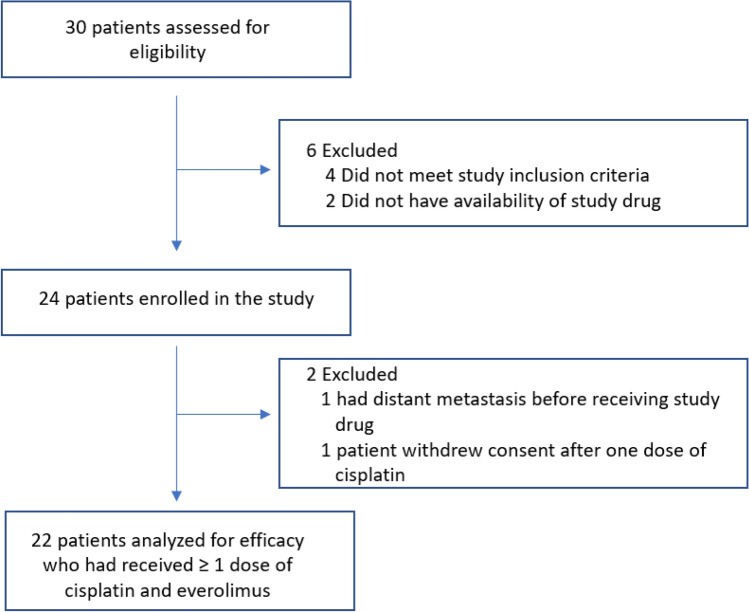
Consort diagram.

Twenty-four patients with triple-negative chemorefractory breast cancer (post anthracycline plus taxane-based chemotherapy, with the biopsy-proven residual disease) were enrolled in the study from June 1, 2013, to August 30, 2017, after obtaining informed written consent (Fig. 1). The median age of the whole cohort was 50.1 years (Table 1). The majority of patients were non-Hispanic Caucasian (66.7%) followed by African American (25%) and Hispanic Caucasian (8.3%). Patients presented with both early-stage and locally advanced disease with 54.2% (13/24) stage II disease while 45.8% (11/24) had stage III disease. Of these twenty-four patients, three had the rare subtype of metaplastic breast cancer (3/24, 12.5%). Patients underwent germline mutation testing, and only 10% (2/20) of those tested had germline genetic mutation, both of which were deleterious PALB2 mutations. Notably, there were no patients with germline BRCA1 or BRCA2 mutations in this chemorefractory TNBC patient cohort. The most common prior standard neoadjuvant regimen received by the study cohort was dose-dense doxorubicin plus cyclophosphamide (60 mg/m^2^ IV doxorubicin plus 600 mg/m^2^ IV cyclophosphamide on Day1 every 14 days × 4 cycles) followed by weekly paclitaxel (80 mg/m^2^ IV every 7 days × 12 cycles).

**Table 1:** Baseline patient characteristics (n = 24).

Median age (range)	50.1 years (31.9–74.4 years)
**Race**	
Non-Hispanic Caucasian	16/24 (66.7%)
African–American	6/24 (25%)
Hispanic Caucasian	2/24 (8.3%)
**Stage prior to treatment (as per AJCC 7th edition)**	
II	13/24 (54.2%)
III	11/24 (45.8%)
**Nodal status prior to treatment**	
N0	8/24 (33.3%)
N1	10/24 (41.7%)
N2	6/24 (25%)
**Genetic testing**	
Performed	20/24 (83.3%)
No germline mutation detected	18/20
Germline mutation detected	2/20 (both had PALB2 mutation)
Not performed	4/24 (16.7%)
**Prior neoadjuvant chemotherapy**	
Dose dense doxorubicin + cyclophosphamide followed by paclitaxel	15/24 (62.5%)
Docetaxel + doxorubicin + cyclophosphamide	7/24 (29.2%)
Ixabepilone + doxorubicin + cyclophosphamide	1/24 (4.2%)
Ixabepilone + cetuximab + doxorubicin + cyclophosphamide	1/24 (4.2%)

### Treatment response

Out of twenty-four patients enrolled, one patient progressed/metastasized prior to receiving the study treatment and a second patient withdrew after 10 days on trial without toxicity or progression, and thus were not evaluated for treatment response. The treatment response was assessed in twenty-two patients. Of these, 10 patients did not complete all four cycles of cisplatin/everolimus—six patients came off the study due to disease progression (one patient developed distant metastasis while five had progression of the primary lesion) and four patients secondary to treatment toxicity. Twenty-one patients out of twenty-two patients underwent definitive surgery. The predefined primary endpoint for these chemorefractory TNBC patients was residual cancer burden (RCB). Sample size estimations were based on historical pathologic response (RCB 0–1) of less than 5%. Here, in this trial, the overall response rate for RCB 0-I was 23% (5/22) (95% confidence interval (CI) 10.1–43.4%). While no patients achieved RCB 0, 5 patients did achieve RCB-I (Table 2). The characteristics of patients achieving RCB 0-I at the surgery are summarized in Table 3.

**Table 2:** Treatment response for patients available for analysis (n = 22).

**Completed all 4 cycles**	
Yes	12
No	10
Progression in primary	5
Progression (metastatic)	1
Toxicity	4
**Definitive surgery**	
Yes	
Breast conservation	5
Mastectomy	16
No	
Metastatic disease progression	1
**Residual cancer burden**	
0–I	5
II–III	16

**Table 3:** Characteristics of patient achieving RCB-I at surgery post cisplatin plus everolimus.

Patient number	Age (years)	Metaplastic	Genetic testing	Residual tumor post neoadjuvant chemotherapy	Actionable mutation	Number of cycles (cisplatin + everolimus)	Tumor at surgery				
							Primary tumor bed			Lymph nodes	
							Primary tumor bed area (mm)	Cancer cellularity (% of area)	% of cancer that is in situ	Number of positive lymph nodes	Diameter of largest metastasis (mm)
1	48	Yes	Not available	1 cm breast mass, 1.4 cm lymph node	PIK3CA mutation (PIK3CA c.3140A>G)	4	4.5 × 2	20	0	0	n/a
7	63	Yes	Negative	2.1 cm	CHEK1 mutation (CHEK1 c.676delA), ATR mutation (ATR c.2320delA), MET mutation (MET c.1039G>A)	4	2 × 1	1	0	0	n/a
8	41	Yes	Negative	2.3 cm	PI3KCA mutation (PI3KCA c.1633G>A)	4	8 × 6	2	0	0	n/a
18	45	No	PALB2 mutated (PALB2 c.1592del)	3.4 cm	No actionable	4	0	0	0	1	3
20	35	No	PALB2 mutated (PALB2 c.3549C>A)	2.3 cm	NOTCH2 mutation	4	10 × 5	5	0	0	n/a

### Survival analysis

**Figure 2:**
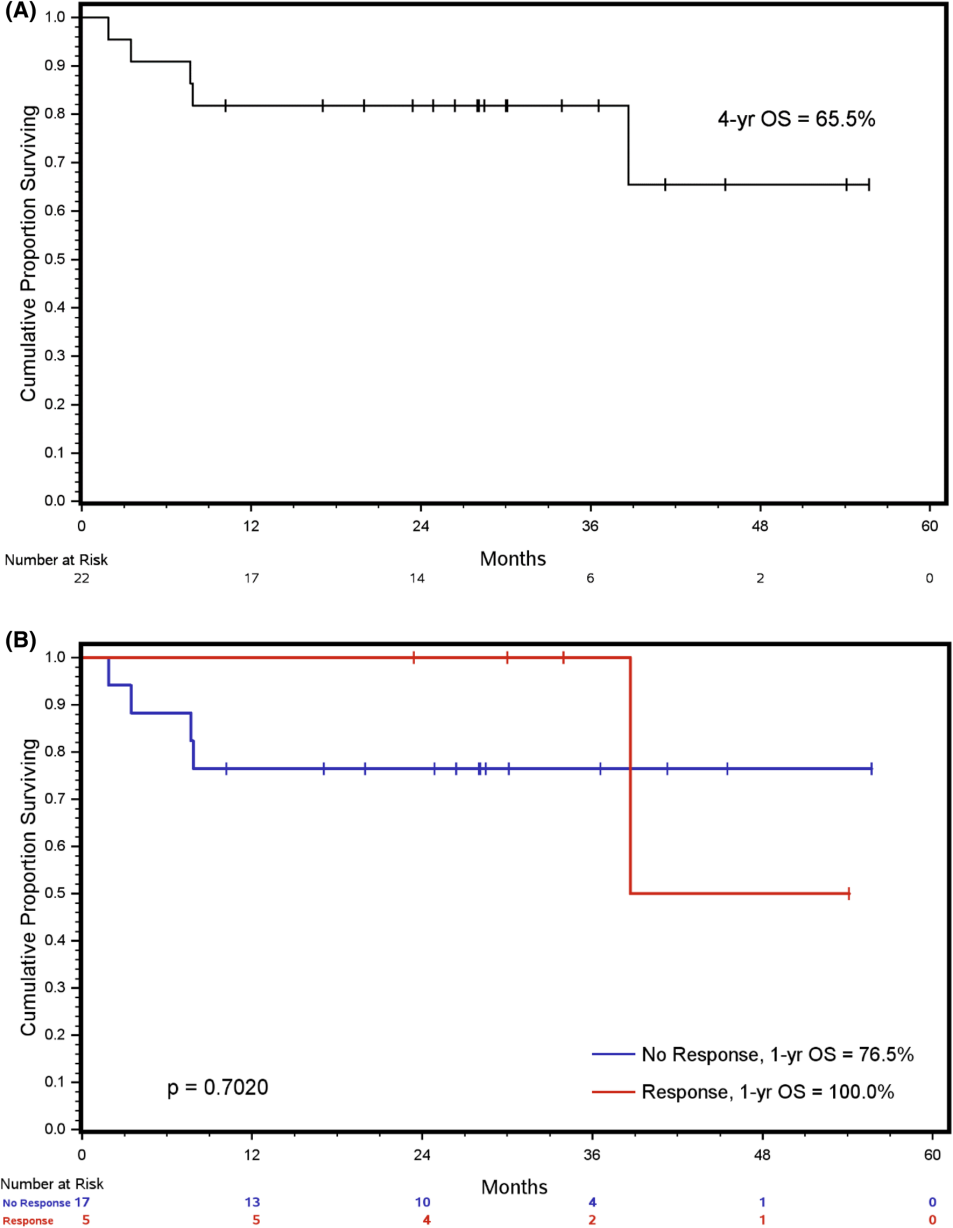
(**A**) OS from the date of first treatment. Estimated OS at 1 year is 81% and 65.5% at 4 years. (**B**) OS in Responders (RCB-I at surgery) vs non-responders (RCB II-III at surgery). (p = 0.7).

At a median follow-up of 29 months (95% CI 25–36.5 months) from study enrollment, 64% (14/22) of the patient remained free of distant metastasis while 46% (6/22) developed metastatic disease. There were 5 deaths observed among the 22 patients. The estimated overall survival (OS) at 1 year was 81% and at 4 years was 65.5%. (Fig. 2A). The 1-year OS was 100% in responders vs. 76.5% in non-responders, which is not statistically significant given the small sample size (Fig. 2B).

### Toxicity

The regimen had fair tolerance with only grade 1 and 2 toxicities in 95.4% (21/22) and grade 3 and 4 in 18.2% (4/22) of the patients. Three main toxicities with incidence > 20% were fatigue (45%), nausea (41%), and mucositis (23%) (see Supplementary Table 1). One patient experienced grade 3 nausea, one patient had both grade 3 thrombocytopenia and grade 3 hyperglycemia, one patient had both grade 3 leucocytopenia and neutropenia while one patient had grade 4 papilledema.

### Next-generation exome sequencing and germline mutation testing

Somatic mutation testing by next-generation exome sequencing was performed in 45% (10/22) of the patients. Somatic mutation testing was performed on pre-treatment biopsy samples using FoundationOne companion diagnostic. Germline mutation testing was available for all these 10 patients, germline testing was done using commercially available Myriad myRisk. Here, no germline BRCA1/2 mutations were detected in patients with triple-negative breast cancer, contrary to expectations. Two patients had germline PALB2 mutations. The most predominant somatic mutation was in *TP53* in 60% of the patients (6/10). Of 5 patients with RCB 0–1, two had deleterious PALB2 germline mutations. All five patients underwent somatic mutation testing. Of these, two patients had actionable somatic PI3KCA mutations. Of interest, of the three patients with metaplastic breast cancer, two patients who had somatic PIK3CA mutation responded with a residual 4 mm (patient #1) and 8 mm (patient #8) tumor post-treatment. On comparing mutation profile between responders and non-responders (see Supplementary Figure 2), mutations affecting PI3K/mTOR pathway and DNA repair mechanisms were enriched in the responder cohort while non-responders had enrichment of *TP53* alterations (all cases). The difference in PALB2 mutation in responders vs. non-responders (p = 0.44) and P1K3CA mutation in responders vs. non-responders (p = 0.44) were not statistically significant while the difference in TP53 alteration in responders vs. non-responders (p = 0.04) was statistically significant using Fisher’s exact test.

## Discussion

In this combination study of everolimus plus cisplatin in the patient’s refractory to standard chemotherapy, where any pathologic response is notable, we report a 23% RCB-I pathologic response. In a long term follow up of patients achieving RCB-0 (pCR) or RCB-I in TNBC post neoadjuvant chemotherapy, prognoses was superior to patients with RCB-II and RCB-III^9^. Relapse-free survival and overall survival were improved in the TNBC patient subset with RCB-0 or RCB-I at surgery. In a multivariate model of relapse-free survival in TNBC, RCB was prognostic independent of other clinical pathologic variables^9^. Except for one patient that developed metastasis while on the study protocol, all others were able to get definitive surgery.

On analysis of characteristics of 5 patients who had RCB-I at surgery, 2 had PALB2 germline mutation, 2 had PIK3CA somatic mutation and one had CHEK1 mutation. PALB2 is a tumor suppressor gene that interacts with BRCA2 and is required for DNA repair^10^. Women with PALB2 mutations are at increased risk of developing breast cancer^11^. More importantly, breast cancer patients with known PALB2 mutations are known to have a poor prognosis^12^. Patients with germline PALB2 mutation may be sensitive to drugs that target DNA repair mechanisms like cisplatin^13^ or PARP inhibitors, which are being tested in clinical trials in various tumor types including pancreatic cancers^14,15^. In our trial, both patients with germline PALB2 mutation had RCB-I at the surgery. Patient #18 with germline PALB2 mutation presented with ~ 10 cm cancer, had large 3.5 cm residual cancer following neoadjuvant anthracyclines and taxanes. Notably, after everolimus and cisplatin, she had no residual cancer in the breast and one small 0.3 cm focus in the axillary node at the time of surgery. The other germline PALB2 mutation carrier (Patient #20) had the chemorefractory disease, with her primary tumor progressing while on standard neoadjuvant anthracycline and taxane. After 4 cycles of everolimus and cisplatin, she responded clinically, and her pathologic residual cancer was under 1 cm. There was no germline BRCA 1 or 2 mutations cancer in this study of chemorefractory patients, reflective of more chemosensitive disease in germline cancers. Two patients who had somatic PI3KCA mutation also responded to cisplatin plus everolimus, both had RCB-I at the surgery. Patient #1 with PI3KCA mutation had only 0.45 cm disease left at surgery post combination therapy, responding even when concomitant *TP53* mutation was present. Patient #8 also had PI3KCA mutation and had less than 1 cm disease at surgery. Of the three patients with metaplastic cancers which are known to be highly refractory to most chemotherapy regimens and to have one of the worst prognosis, two patients (Patient #1 and #8) who had PIK3CA mutation were able to achieve RCB-I at the surgery. The remaining one metaplastic patient (#7) did not have PIK3CA mutations but the CHEK1-ATR pathway mutation also achieved RCB-I at the surgery.

The overall survival of the whole cohort was 81% at 1 year and 65.5% at 4 years. Estimated OS at 1 year was higher in patients achieving RCB-I vs. others at 100% vs. 76.5% respectively, although this was not statistically different due to a low number of patients in this trial. Patients in the responder group had enrichment of mutation affecting DNA repair (PALB2, CHEK1, and ATR) and PI3KCA/mTOR pathway, compared to non-responders. Non-responders had enrichment of TP53 alterations compared to responders.

Everolimus has been previously studied in combination with chemotherapy in the upfront neoadjuvant setting in TNBC where its addition led to a lower pCR rate compared to chemotherapy with increased toxicity^16^. In this trial, the regimen consists of everolimus in combination with cisplatin, after neoadjuvant chemotherapy where the residual tumors have been shown to have an enrichment of both the PI3K/mTOR pathway along with DNA repair alterations as the major mechanistic pathways of chemotherapy resistance^8^. We report a noteworthy 23% of the patients achieved RCB-I at the surgery. In this trial, patients who had mutations affecting the DNA repair pathway and patients with somatic PI3KCA mutations had a response to treatment with cisplatin plus everolimus. There are limitations of this study, namely a low number of patients and a single-arm study with no randomization arm. Despite the limitations, these results add to the understanding of targeting the PI3K/mTOR pathway in TNBC especially in patients who have residual disease post neoadjuvant chemotherapy where the current standard of care is the use of adjuvant capecitabine^17^. Further studies evaluating cisplatin plus everolimus in cohorts with PI3K pathway alteration or PALB2 germline mutation is needed especially in poor prognosis metaplastic breast cancer patients^18^.

## Conclusion

The combination of everolimus plus cisplatin is active in the neoadjuvant setting in TNBC patients who have residual disease post standard neoadjuvant chemotherapy with a response rate of 23% for RCB-I at the surgery. This combination was active in a subset of patients with germline PALB2 mutation or somatic PI3KCA mutation. This is the first study using targeted therapy in a neoadjuvant setting for TNBC patients who had residual disease post standard anthracycline-taxane neoadjuvant chemotherapy. Responders to this combination included patients with germline PALB2 mutation and metaplastic histology who are known historically have a poor prognosis. Further studies evaluating this regimen in cohorts with PI3KCA mutation or PALB2 germline mutation is needed.

## Patients and methods

### Patients

All female patients > 18 years of age with TNBC defined as estrogen receptor-negative and progesterone receptor-negative (< 10% staining by immunohistochemistry (IHC) for estrogen and progesterone receptor) plus HER2 negative (FISH ratio < 2.2 or IHC 0–1 + or IHC 2–3 + and FISH ratio < 2.2) who had clinical and pathological documentation of residual disease of > 1 cm after neoadjuvant chemotherapy were eligible for the trial (Clinicaltrials.gov identifier: NCT01931163. Registered on 29/08/2013). Trial was approved by Houston Methodist Hospital Institutional Review Board (IRB) and monitored by the Houston Methodist Hospital Data Safety and Monitoring Board (DSMB). All methods were performed in accordance with the relevant guidelines and regulations. Written informed consent was obtained from all patients before sample and data collection. Patients had to be Eastern Cooperative Oncology Group (ECOG) performance status 0 or 1. Exclusion criteria included women who were pregnant or breastfeeding, history of malabsorption syndrome, allergy to everolimus or other rapamycin analogs, and previous cancer (except for non-melanoma skin cancer or cervical carcinoma in situ) in the past 5 years.

### Study design and treatment plan

Patients with TNBC received standard neoadjuvant anthracycline and taxane-based chemotherapy regimen and had to have the biopsy-proven residual disease before being eligible. Prior to enrollment to this clinical study, all patients underwent baseline biopsy from a primary tumor or an abnormal axillary lymph node. Everolimus was given daily orally at 10 mg for 12 weeks and cisplatin was given intravenously at 20 mg/m^2^ on days 1, 8, 15 for 4 cycles (each cycle of 21 days). Patients then underwent definitive surgery, including axillary lymph node dissection, if indicated. The specimen was evaluated for chemotherapy response. RCB analysis was performed on the specimen by a previously validated method^19^. Toxicity was assessed by Common Terminology Criteria for Adverse Events (CTCAE) version 3.0 (see Supplementary Figure 1).

### Statistical methods

Median follow-up was estimated by the reverse Kaplan–Meier method^20^. Survival was estimated using the Kaplan–Meier method and 95% confidence intervals for timepoint survival probabilities (e.g., 1-year survival) were calculated using a log–log transformed pointwise method. The Wilson score method was used to construct 95% confidence intervals for the response. All analyses were conducted using SAS 9.4 (SAS Institute Inc., Cary, NC, USA).

### Ethics approval and consent

Trial was monitored by the institutional Data Safety and Monitoring Board (DSMB). Written informed consent was obtained from all patients before sample and data collection. The trial was registered at Clinicaltrials.gov identifier: NCT01931163. Registered August 29th, 2013. https://clinicaltrials.gov/ct2/show/NCT01931163.

### Animal subjects

This article does not contain any studies with animals performed by any of the authors.

## Supplementary Information

Supplementary Information.

## Data Availability

The datasets supporting the conclusions for the current study are stored in a secured shared drive and will be shared by the corresponding author upon reasonable request.
