# Supplementary material for: Targeting mTOR and DNA repair pathways in residual triple negative breast cancer post neoadjuvant chemotherapy
Source: Sci Rep. 2021 Jan 8;11:82. doi: 10.1038/s41598-020-80081-y (PMC7794349; doi:10.1038/s41598-020-80081-y)
Supplement: Supplementary file 1 — Supplementary Information. [file 41598_2020_80081_MOESM1_ESM.docx]

Targeting mTOR and DNA Repair Pathways in Residual Triple Negative Breast Cancer post Neoadjuvant Chemotherapy

Authors: Kartik Anand^1^, Tejal Patel^1^, Polly Niravath^1^, Angel Rodriguez^1^, Jorge Darcourt^1^, Anna Belcheva^1^, Toniva Boone^1^, Joe Ensor^2^, Jenny Chang^1,2^

1. Houston Methodist Cancer Center/Weill Cornell Medicine, Houston, TX 77030
2. Houston Methodist Research Institute, Houston, TX 77030

Corresponding Author:

Jenny C. Chang, MBChirB, MD

Emily Hermann Chair in Cancer Research

Director, Houston Methodist Cancer Center/Weill Cornell Medicine

OPC 24, 6445 Main Street,

Houston, TX 77030

Email: [jcchang@houstonmethodist.org](mailto:jcchang@houstonmethodist.org)

Telephone: 713-441-9948

Word count: 2138

Running title: Neo-adjuvant Everolimus plus Cisplatin for TNBC patients

Supplementary:

Figure 1: Study protocol.

Figure 2: Mutation analysis of responders vs. non-responders.

Table 1: Toxicities with incidence >20%

Figure 1:

**
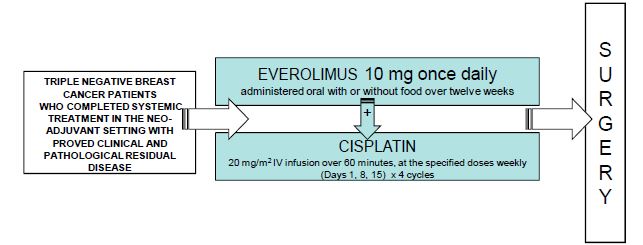
**

Figure 1. Study protocol. Primary objective of the trial was rate of pathologic response, as measured by Residual Cancer Burden (RCB) after treatment with cisplatin plus everolimus

Figure 2:


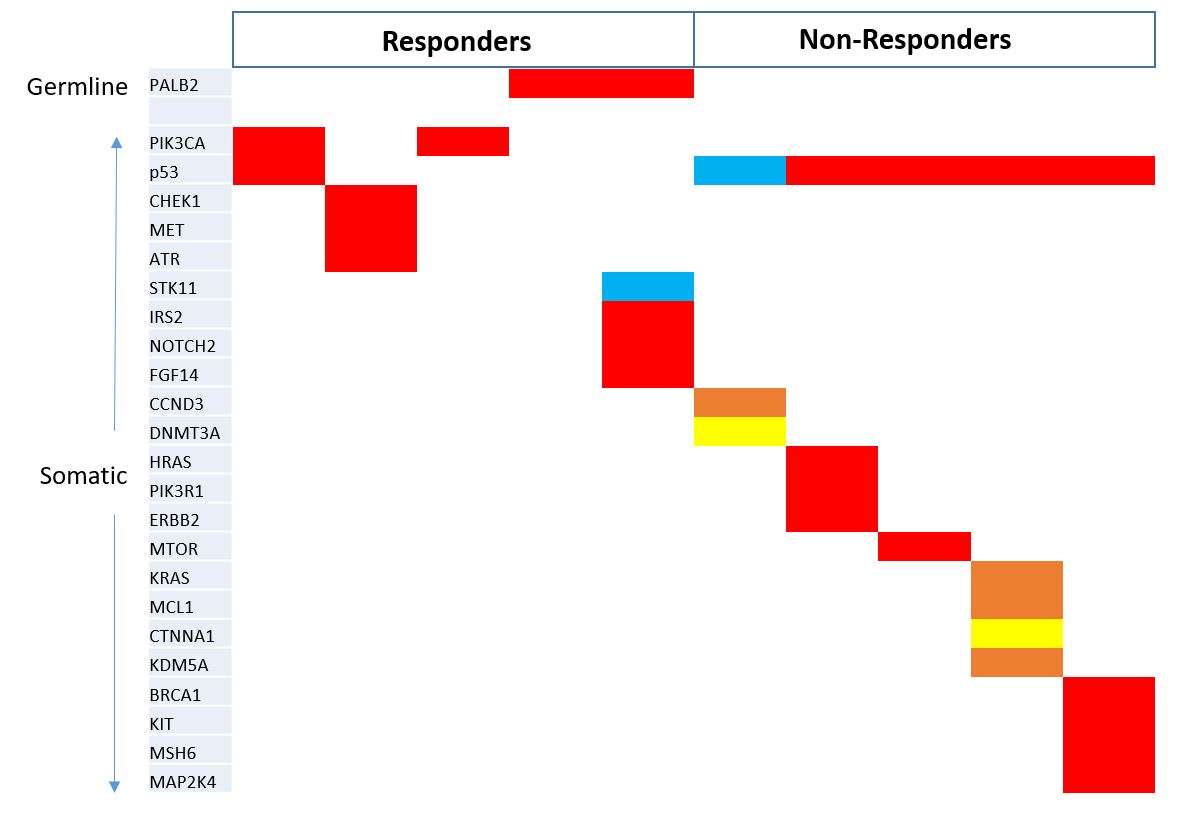


Figure 2. Analysis of germline mutations and somatic mutations (by targeted exome next generation sequencing) between responders vs. non-responders.

Red=mutations, Blue=variants, , Yellow=copy number loss, Orange=amplification

**Table 1.** Toxicities with incidence >20%

| **Toxicity** | **% of patients** |
| --- | --- |
| Fatigue | 45% (10/22) |
| Nausea | 41% (9/22) |
| Mucositis | 23% (5/22) |
